# A study on the relationship between research stress, research anxiety, research performance, and job satisfaction among Chinese healthcare professionals and its influencing mechanisms: a national multi-center survey

**DOI:** 10.3389/fpsyt.2026.1894224

**Published:** 2026-07-16

**Authors:** Yuanlong Huang, Chunsong Yang, Siyi He

**Affiliations:** 1 Department of Gastroenterology,Chengdu Xinhua Hospital Affiliated to North Sichuan Medical College, Chengdu, Sichuan, China; 2 Department of Pharmacy, Evidence-based Pharmacy Center, West China Second Hospital, Sichuan University, Chengdu, Sichuan, China; 3 Key Laboratory of Birth Defects and Related Diseases of Women and Children (Sichuan University), Ministry of Education, Chengdu, Sichuan, China; 4 School of Public Health, Gansu University of Chinese Medicine, Lanzhou, Gansu, China

**Keywords:** healthcare professionals, job satisfaction, research anxiety, research performance, research stress

## Abstract

**Background:**

Chinese healthcare professionals face dual pressures from clinical duties and research activities, which may increase research-related anxiety and reduce job satisfaction. While occupational stress has been well studied, the mechanisms linking research stress to job satisfaction are unclear. Research performance may also play a key role, but its impact remains uncertain. This study explores the relationships between research stress, research anxiety, research performance, and job satisfaction, and identifies the pathways connecting these variables.

**Methods:**

A cross-sectional survey was conducted among healthcare professionals (nurses, physicians, and pharmacists) from eastern, central, and western regions of China to assess research-related conditions and job satisfaction (JS). Job satisfaction was measured using a modified validated scale. Research stress (RS), research anxiety (RA), and research performance (RP) were assessed using self-developed instruments. Structural equation modeling (SEM) was employed to perform path analysis and mediation analysis.

**Results:**

A total of 924 healthcare professionals were included in the study. Over 85% reported working more than 40 hours per week, and 66.5% rated their health status as fair or poor. Regarding the types of research conducted by healthcare professionals, clinical research accounted for the highest proportion (63.2%), followed by basic science research (9.8%), while health services research and community-based research were relatively less common. The mean scores of JS, RS, RA, and RP were 3.40 ± 0.88, 2.56 ± 0.70, 2.34 ± 0.87, and 3.55 ± 0.82, respectively. Path analysis revealed that research stress was positively associated with research anxiety and negatively associated with both research performance and job satisfaction. Research anxiety was also negatively associated with research performance and job satisfaction, whereas research performance was positively associated with job satisfaction. Mediation analysis indicated that research stress was associated with job satisfaction both directly and indirectly through research anxiety and research performance.

**Conclusions:**

Research stress is negatively associated with job satisfaction among healthcare professionals through increased research anxiety, whereas higher research performance is positively associated with job satisfaction. Hospitals and healthcare institutions should optimize the research environment, strengthen psychological support, and enhance research resource allocation to reduce research stress, improve research performance, and ultimately increase job satisfaction.

## 1 Background

With the continuous growth in healthcare demands, healthcare professionals—particularly nurses, physicians, and pharmacists—are facing increasingly severe work-related stress and mental health challenges, which have become critical issues for healthcare systems worldwide (1–4). In China, healthcare professionals are not only responsible for clinical care, treatment, and pharmaceutical services but are also increasingly required to engage in medical research, thereby serving as key contributors to hospital research productivity. Previous studies have shown that although most clinical healthcare professionals participate in research-related activities, their overall research literacy remains suboptimal, and their research competence and academic writing skills require further improvement (5–7). However, escalating research demands—especially within high-intensity clinical environments—may be negatively associated with research performance and, in turn, with job satisfaction (8).

### 1.1 Sources of research stress and its impact on anxiety

Research stress refers to the psychological tension experienced by healthcare professionals while completing research-related tasks. Such stress arises from multiple sources. First, healthcare professionals must allocate time between demanding clinical responsibilities and research obligations, the latter often accompanied by high expectations and performance standards. Second, limited time and institutional resources may hinder effective research engagement. According to the Job Demands–Resources (JD-R) model, excessive job demands that exceed available resources are positively associated with strain and anxiety, which in turn are negatively associated with work performance (9, 10). Empirical evidence suggests a significant positive association between research stress and research anxiety (11). Increased anxiety is frequently accompanied by concerns about research outcomes and fear of failure, which further intensify psychological burden and reduce work efficiency and mental well-being (11).

### 1.2 The relationship between research stress and research performance

Excessive research stress may not only elevate anxiety but also impair research performance by diverting attention and depleting cognitive resources. The Yerkes–Dodson law posits that moderate levels of stress may be positively associated with motivation and performance, whereas excessive stress may be negatively associated with concentration and work quality (12).For healthcare professionals, conflicts between clinical duties and research tasks may prevent full engagement in research activities, thereby compromising the quality of research outputs (13). Consequently, excessive research stress may weaken research performance and limit academic productivity.

### 1.3 The relationship between research anxiety and research performance

Research anxiety, as a variable closely associated with research stress, has been widely shown to be negatively associated with performance. According to attentional control theory, elevated anxiety disrupts working memory, decision-making ability, and creative thinking (14, 15). When healthcare professionals experience anxiety, cognitive resources are consumed by intrusive worries, reducing their ability to concentrate on research tasks and lowering work efficiency (14–16).

Beyond impairing research quality, anxiety may also contribute to professional burnout, thereby influencing job satisfaction.

### 1.4 The relationship between research performance and job satisfaction

Research performance is positively associated with job satisfaction. Based on social identity theory (17), recognition of professional achievements enhances individuals’ sense of accomplishment and social identity. Successful research outcomes may elevate healthcare professionals’ professional status and recognition within their field, thereby strengthening occupational engagement and satisfaction. Conversely, inadequate research support or excessive research pressure may limit perceived career development opportunities, leading to dissatisfaction and reduced job satisfaction.

### 1.5 The mediating role of research anxiety in the relationship between research stress and research performance

Research stress may be indirectly associated with research performance through increased anxiety. Excessive stress typically triggers anxiety, which consumes cognitive and psychological resources necessary for effective task performance (13, 18, 19). Anxiety may reduce focus and work efficiency, thereby impairing research performance. Additionally, sustained anxiety may weaken professional identity and work attitudes, further diminishing performance outcomes.

### 1.6 Research stress and job satisfaction

Job satisfaction, as a key indicator of work attitudes, is associated with multiple factors. (20–22). Excessive research stress may contribute to burnout among healthcare professionals, thereby directly reducing job satisfaction. According to the JD-R model (23), when job demands exceed available psychological and temporal resources, stress and burnout increase, ultimately decreasing job satisfaction. Thus, high levels of research stress may be negatively associated with work motivation and positively associated with negative professional emotions, which in turn are negatively associated with overall job satisfaction.

Although existing research has extensively examined occupational stress, anxiety, mental health, and burnout among healthcare professionals (4, 24–27), systematic investigations into how research stress is associated with research performance and job satisfaction remain limited. Most prior studies have focused on the relationship between general work stress and mental health. While some research has explored healthcare professionals’ research participation (5–7, 28), few studies have examined the integrated relationships among research stress, research anxiety, research performance, and job satisfaction.

### 1.7 Theoretical framework and mechanism pathway

This study adopts the Job Demands–Resources (JD-R) model as the core theoretical framework to systematically explain the relationships among research stress, research anxiety, research performance, and job satisfaction. According to the JD-R model, when job demands exceed available resources, psychological strain and anxiety are triggered, which in turn can impair work performance and mental well-being. In this study, research stress is regarded as a specific type of job demand that consumes healthcare professionals’ cognitive and temporal resources, thereby increasing research anxiety. Research anxiety can weaken attention, decision-making ability, and creative thinking, leading to reduced research performance. The decline in research performance is subsequently negatively associated with job satisfaction, as the quality of research outcomes and a sense of professional achievement are key factors related to higher job satisfaction. The mechanism can be summarized as the following pathway:

Research stress → Research anxiety → Research performance → Job satisfaction

(1) Research stress: The pressure associated with balancing clinical duties and research tasks. (2) Research anxiety: Psychological tension arising from concerns over research outcomes, fear of failure, and constraints in time and resources. (3) Research performance: The quality and efficiency of research outputs, related to cognitive and emotional resources. (4) Job satisfaction: Healthcare professionals’ subjective evaluation of their professional achievement and recognition.

**Figure 1:**
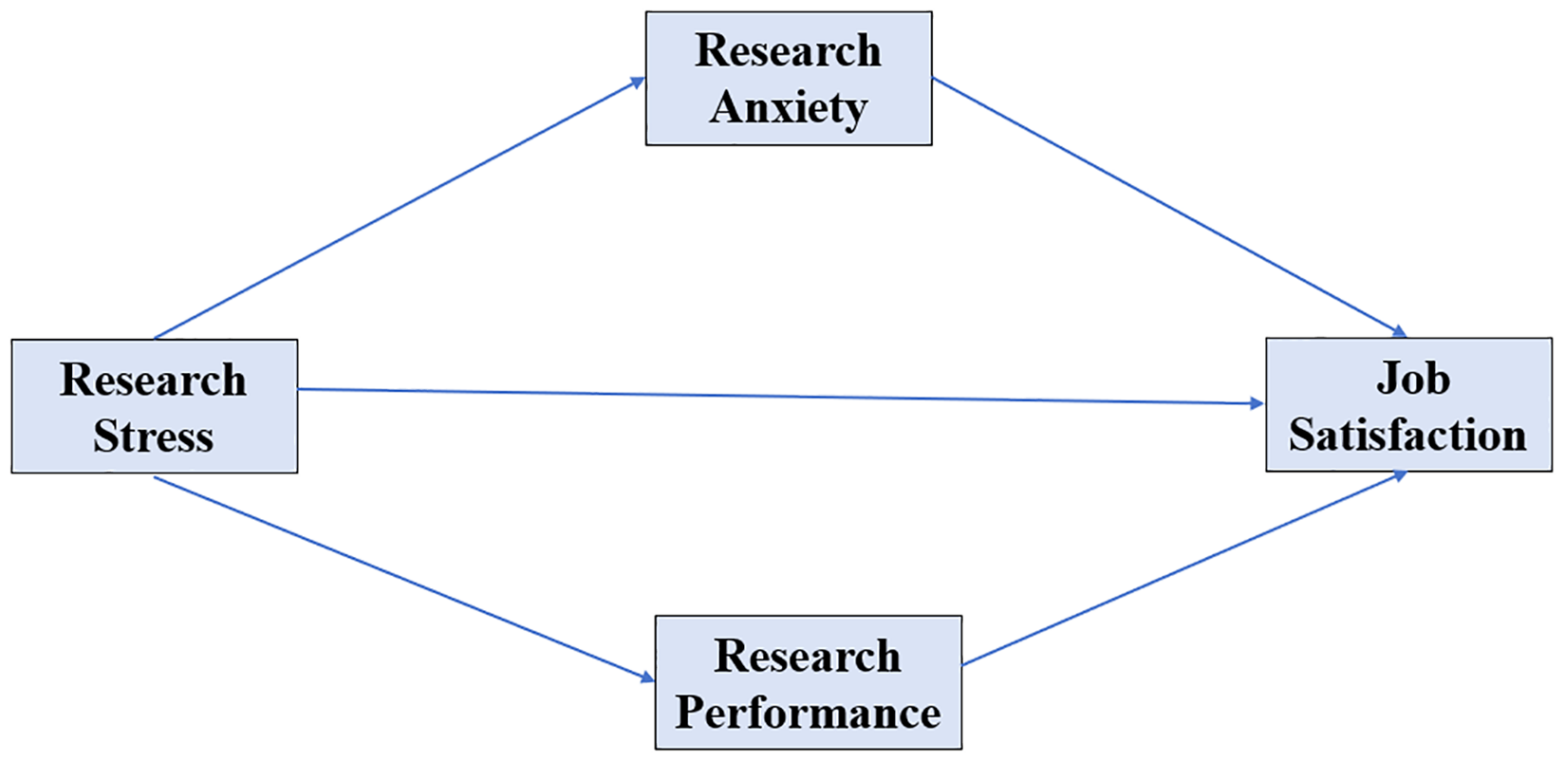
Conceptual model of the association of research stress with job satisfaction via research anxiety and research performance.

As illustrated in **Figure 1**, research anxiety and research performance act as sequential mediators between research stress and job satisfaction. This conceptual model provides theoretical support for understanding how research-related demands are associated with healthcare professionals’ research performance and overall job satisfaction.

Existing literature primarily addresses the associations between research stress and research output, with limited exploration of the mechanisms through which research stress is associated with job satisfaction via anxiety and performance. Guided by the JD-R framework (9, 10), excessive job demands may impair both performance and psychological well-being when resources are insufficient. However, most studies emphasize clinical workload stress (27, 29), with comparatively little attention given to research-related stress.

Although research anxiety has been shown to be negatively associated with performance, its mediating role between research stress and job satisfaction has not been systematically examined. Therefore, this study integrates research stress, research anxiety, research performance, and job satisfaction into a unified framework to examine how research stress is associated with performance through anxiety and is ultimately associated with job satisfaction. The findings provide both theoretical and empirical evidence for optimizing the research environment, alleviating research stress, and enhancing research performance and job satisfaction among healthcare professionals.

## 2 Methods

### 2.1 Study design

This study employed a cross-sectional survey design to investigate research stress, research anxiety, research performance, and job satisfaction among healthcare professionals working in hospitals across eastern, central, and western China. Quantitative data were collected by structured questionnaires, and structural equation modeling (SEM) was applied to examine the relationships among the study variables.

### 2.2 Participants

#### 2.2.1 Inclusion criteria

Participants were required to meet the following criteria: (1) aged between 20 and 60 years; (2) healthcare professionals, including nurses, physicians, and pharmacists; (3) currently engaged in clinical practice as formally employed staff members; and (4) provided written informed consent.

#### 2.2.2 Exclusion criteria

Participants were excluded if they had severe psychological or physical disorders;

### 2.3 Sample size and sampling procedure

Based on the information you provided, we use the sample size calculation formula: $n\;=\;{\left(\frac{Z_{\alpha /1}\cdot \sigma }{\delta }\right)}^{2}$ to estimate the required minimum sample size. Assuming the reported satisfaction standard deviation is 0.991 (30), the allowable error δ is 0.1, and the confidence level is 95%. Therefore, the calculated sample size is 378. To account for attrition or incomplete surveys, the sample size is typically increased by 10%. Thus, the adjusted sample size is 416. This ensures the reliability of the research results and accounts for potential attrition.

A convenience sampling method was adopted. Healthcare professionals (nurses, physicians, and pharmacists) from hospitals in eastern, central, and western regions of China were invited to participate in the study. Data collection was conducted from October 2024 to March 2025.

### 2.4 Data collection

#### 2.4.1 Baseline data collection

Data were collected through an online questionnaire. Information included sociodemographic characteristics, research stress, research anxiety, research performance, and job satisfaction. Baseline variables included: Demographic characteristics (gender, age, professional position, marital status, number of children); Educational background (degree level and academic qualifications); Professional information (professional title, hospital level, years of work experience); Work characteristics (weekly working hours and proportion of time devoted to research); Self-rated health status; Geographic region (eastern, central, or western China).

#### 2.4.2 Assessment of research status

The survey assessed research-related stress, anxiety, performance, and overall job satisfaction.

The scale was developed based on a thorough review of relevant literature on work-related stress, anxiety, performance, and job satisfaction in healthcare settings. Experts in healthcare research were consulted to ensure the items adequately captured the various aspects of these constructs, providing a comprehensive measurement of the factors influencing research stress and related outcomes.

#### 2.4.3 Measurement instruments

##### 2.4.3.1 Research stress scale

The Research Stress Scale was self-developed to measure research-related stress among healthcare professionals. The scale consists of 13 items across four dimensions: (1) Task Pressure (TP), (2) Innovation Pressure (IP), (3) Cooperation and Communication Pressure (CCP), (4) Resource and Career Development Pressure (RPCD). Items were rated on a 5-point Likert scale, with higher scores indicating greater research stress. Cronbach’s α values of these four dimensions ranged from 0.808 to 0.872. The absolute values of the standardized factor loadings for each factor are greater than 0.7 and are statistically significant, indicating a strong measurement relationship.

##### 2.4.3.2 Research anxiety scale

The Research Anxiety Scale was self-developed to assess anxiety experienced during research activities. The scale contains four items evaluating anxiety-related feelings during research tasks. Responses were measured using a 5-point Likert scale, with higher scores indicating higher levels of research anxiety. Cronbach’s α values of RA was 0.856. The standardized factor loadings for each factor exceed 0.7 and are significant, indicating a strong measurement relationship.

##### 2.4.3.3 Research performance scale

The Research Performance Scale was self-developed to evaluate participants’research engagement and outcomes. The scale includes four items rated on a 5-point Likert scale, with higher scores representing better research performance.

Cronbach’s α values of RP was 0.821. Standardized factor loadings above 0.7 and their significance suggest a robust measurement relationship.

##### 2.4.3.4 Job satisfaction scale

Job satisfaction was assessed using a modified version of the Job Satisfaction Scale (31). The scale includes six items measuring overall satisfaction with the medical profession and current employment status. Items were rated on a 5-point Likert scale (1 = very dissatisfied; 5 = very satisfied), with higher scores indicating greater job satisfaction. Cronbach’s α values of JS was 0.899. The factor loadings greater than 0.7 and their statistical significance reflect a solid measurement relationship.

### 2.5 Hypothesized model

Based on theoretical frameworks and prior research, the following hypotheses were proposed:

There is a relationship among research stress, research anxiety, research performance, and job satisfaction. Specifically, research stress is positively related to research anxiety, and negatively related to research performance; research anxiety is negatively related to research performance; and research performance is positively related to job satisfaction.

### 2.6 Data analysis

Data analysis was performed using SPSS version 26.0 and AMOS version 24.0. The analytical procedures included:

(1)Descriptive Statistics: Means, standard deviations, and proportions were calculated to describe baseline characteristics and study variables. (2)Reliability and Validity Testing: Internal consistency of the scales was assessed using Cronbach’s alpha coefficients, with values above 0.70 indicating acceptable reliability. Construct validity was examined through both exploratory factor analysis (EFA) and confirmatory factor analysis (CFA). For EFA, principal component analysis with varimax rotation was used to explore the factor structure. For CFA, model fit was evaluated using multiple indices: CMIN/DF (<3 for excellent fit, <5 acceptable), RMR (<0.05 excellent, <0.08 acceptable), GFI, NFI, TLI, and CFI (>0.90 excellent, >0.80 acceptable), and RMSEA (<0.08 excellent, <0.10 acceptable). Standardized factor loadings above 0.60 with statistical significance (p < 0.001) were considered evidence of good measurement validity. Standardized path coefficients were examined to assess the direct and indirect effects of research stress, research anxiety, and research performance on job satisfaction. Mediation analysis was conducted to determine whether research anxiety mediated the relationship between research stress and research performance, and whether research anxiety and research performance jointly mediated the relationship between research stress and job satisfaction.

### 2.7 Ethical considerations

This study was approved by the Ethics Committee of West China Second Hospital, Sichuan University (Approval No. 2025218). All procedures complied with ethical standards for research involving human participants. Written informed consent was obtained from all participants prior to data collection. The study adhered to ethical principles, including respect for participants’ rights, protection of privacy, and voluntary participation.

## 3 Results

### 3.1 Characteristics of research participants

A total of 1002 participants were initially recruited for the study, of whom 78 declined to participate. Therefore, 924 participants from the eastern, western, and central regions of China were included in the study. Among them, doctors accounted for 38.2% (353 participants), pharmacists for 28.8% (266 participants), and nurses for 33.0% (305 participants). The majority held bachelor’s degrees (42.3%) and master’s degrees (33.9%). Over 85% of the participants worked more than 40 hours per week. 66.5% of the participants reported that their health status was average or poor. Regarding the time spent on research work, 41.1% of the participants stated they spent less than 10% of their weekly time on research, 27.1% spent 11%-25%, and 31.1% spent 26%-50% of their weekly time on research. In terms of research type, clinical research accounted for the largest proportion (63.2%), followed by basic scientific research (9.8%), while health services research and community research had relatively low proportions (**Table 1**). The scores for JS, RS, RA and RP were 3.402 ± 0.877, 2.561 ± 0.697, 2.338 ± 0.871 and 3.549 ± 0.823.

**Table 1:** Basic characteristics of the study subjects.

Variables	N(%)	Variables	N(%)
Gender		Education level	
Male	270(29.2%)	Associate degree or below	82(8.9%)
Female	654(70.8%)	Bachelor’s degree	391(42.3%)
Region		Master’s degree	313(33.9%)
Western region	323(35.0%)	Doctoral degree	138(14.9%)
Central region	225(24.4%)	Weekly working hours	
Eastern region	376(40.7%)	40 hours or less	132(14.3%)
Hospital level		41-50 hours	435(47.1%)
Tertiary	819(88.6%)	51-60 hours	326(35.3%)
Secondary	105(11.4%)	61 hours or more	31(3.4%)
Age		Proportion of research time	
<30 years	260(28.1%)	Less than 10%	380(41.1%)
31 to 40 years	399(43.2%)	11%-25%	250(27.1%)
>40 years	265(28.7%)	26%-50%	287(31.1%)
Professional title		More than 50%	8(0.7%)
Junior	149(16.1%)	Type of research	
Intermediate	267(28.9%)	Clinical research	584(63.2%)
Associate senior	238(25.8%)	Basic scientific research	91(9.8%)
Senior	270(29.2%)	Translational research	207(22.4%)
Marital status		Health services research	24(2.6%)
Unmarried	305(33.0%)	Community-based research	2(0.2%)
Married	565(61.1%)	Educational research	8(0.9%)
Divorced	36(3.9%)	Economic research	8(0.9%)
Widowed	18(1.9%)	Health status	
Children status		Very healthy	106(11.5%)
No children	341(36.9%)	Fairly healthy	213(23.1%)
One child	431(46.6%)	Average	229(24.8%)
Two or more children	152(16.5%)	Not very healthy	209(22.6%)
		Very unhealthy	167(18.1%)

### 3.2 Reliability and validity analysis

All scales demonstrated strong internal consistency, with Cronbach’s α values ranging from 0.808 to 0.899 (**Supplementary Table 1**). The cumulative variance explained by the 7 common factors was 70.439%, and all factor loadings exceeded 0.4, indicating robust structural validity (**Supplementary Tables 2**, **3**).To examine the construct validity of the measurement instruments, confirmatory factor analysis (CFA) was conducted for the scales of research stress, research anxiety, research performance, and job satisfaction. The factor loadings indicated that all items had significant and appropriate relationships with their corresponding latent variables. Specifically, the standardized factor loadings for the six job satisfaction (JS) items ranged from 0.750 to 0.808; for the thirteen research stress (RS) items across four dimensions, the loadings ranged from 0.733 to 0.822; the four research anxiety (RA) items had loadings ranging from 0.756 to 0.797; and the four research performance (RP) items had loadings between 0.714 and 0.758. All factor loadings exceeded 0.6 in absolute value and were statistically significant (p < 0.001), indicating that the items effectively measured their respective latent constructs (**Supplementary Table 4**). Overall, the results of the confirmatory factor analysis demonstrate that the scales used in this study possess good construct validity and measurement reliability, providing a solid basis for subsequent structural equation modeling analyses. The average variance extracted (AVE) was above 0.5, supporting convergent validity (**Supplementary Table 1**).

### 3.3 Correlation analysis

Job Satisfaction was significantly positively correlated with Research Performance (r = 0.354) (p < 0.01), Job Satisfaction was significantly negatively correlated with Research Stress (r = -0.430) and Research Anxiety (r = -0.422) (p < 0.01). Detailed data are shown in (**Supplementary Table 5**).

### 3.4 Path and mediation effect analysis

The structural equation model showed a good fit based on the fit indices (**Supplementary Table 6**). The path analysis results indicated (**Table 2**) that Research Stress was significantly positively associated with Research Anxiety (standardized path coefficient = 0.584, P < 0.05), and significantly negatively associated with Research Performance (standardized path coefficient = -0.425, P < 0.05) and Job Satisfaction (standardized path coefficient = -0.314, P < 0.05). Research Anxiety was significantly negatively associated with Research Performance (standardized path coefficient = -0.228, P < 0.05) and Job Satisfaction (standardized path coefficient = -0.241, P < 0.05). Research Performance was significantly positively associated with Job Satisfaction (standardized path coefficient = 0.117, P < 0.05). Mediation analysis (**Table 3**) showed that Research Stress was directly negatively associated with Job Satisfaction, and was also indirectly associated with Job Satisfaction through Research Anxiety and Research Performance.

**Table 2:** Path analysis results.

Path analysis			Estimate	S.E.	C.R.	P	STD estimate
RA	<-–	RS	0.81	0.067	12.161	***	0.584
RP	<-–	RS	-0.535	0.069	-7.758	***	-0.425
RP	<-–	RA	-0.207	0.043	-4.798	***	-0.228
JS	<-–	RA	-0.256	0.048	-5.304	***	-0.241
JS	<-–	RS	-0.463	0.08	-5.777	***	-0.314
JS	<-–	RP	0.138	0.052	2.636	0.008	0.117

JS, Job Satisfaction; RS, Research Stress; RA, Research Anxiety; RP, Research Performance, ***p < 0.01.

**Table 3:** Mediation effect test.

Effect types	Parameter	Estimate	Lower	Upper	P
Direct effect	RS→JS	-0.463	-0.651	-0.296	<0.001
Indirect effect	RS→RA→JS	-0.207	-0.301	-0.126	<0.001
	RS→RP→JS	-0.074	-0.147	-0.017	0.011
	RS→RA→RP→JS	-0.023	-0.052	-0.005	0.01
Total effect	RS→JS	-0.767	-0.93	-0.624	<0.001

## 4 Discussion

### 4.1 Main findings and interpretation

This nationwide multicenter, cross-sectional study aimed to examine the associations among research stress, research anxiety, research performance, and job satisfaction among healthcare professionals. As this study employed a cross-sectional design, the findings reflect associations between variables and do not allow for causal inferences. The findings indicate that research stress is significantly positively associated with research anxiety and is indirectly associated with research performance through anxiety, which in turn is negatively associated with job satisfaction.

Specifically, research stress was positively associated with research anxiety, which in turn was negatively associated with research performance and job satisfaction. These findings are consistent with previous studies (11, 13, 32–34). Under conditions of high work demands, healthcare professionals are more likely to experience elevated anxiety, which may impair work efficiency and hinder research productivity. Furthermore, this study demonstrated that research stress is indirectly associated with research performance through research anxiety and is directly negatively associated with job satisfaction. This suggests that a high-pressure research environment may contribute to occupational burnout and weaken professionals’ identification with and satisfaction toward their careers.

In addition, this study found a positive association between research performance and job satisfaction, but this relationship is not merely a simple outcome. From a mechanistic perspective, high levels of research performance may enhance job satisfaction through multiple pathways. First, from the perspective of cognitive resource depletion, higher research performance indicates that healthcare professionals can efficiently manage their time and attentional resources, reducing the cognitive burden caused by task failure or anxiety, thereby maintaining greater focus and task execution at work. Second, from the perspective of emotional regulation, successfully completing research tasks can enhance positive emotions, alleviate work-related stress, and improve psychological resilience, thereby improving overall job satisfaction. Finally, from the perspective of professional identity, higher research performance increases a sense of professional achievement and social recognition, strengthening individuals’ identity within their professional community, which encourages greater engagement and leads to higher occupational satisfaction.

In summary, research performance not only reflects objective research outputs but also indirectly promotes overall job satisfaction among healthcare professionals through mechanisms such as cognitive resource management, emotional regulation, and professional identity. This mechanistic analysis provides deeper theoretical support for understanding the complex relationships between research stress and job satisfaction.

### 4.2 Comparison with previous studies

The findings of this study are consistent with prior international research. For example, Khamisa et al. (35) reported that high-pressure work environments are positively associated with anxiety among healthcare professionals, which in turn is negatively associated with their psychological well-being and job performance. Similarly, numerous studies have demonstrated that work-related stress and anxiety are significantly associated with reduced job satisfaction among healthcare professionals (1, 36).

However, the present study extends existing literature by employing structural equation modeling to systematically examine the underlying mechanisms linking research stress and job satisfaction. Beyond confirming the direct impact of research stress on anxiety, our findings further demonstrate the mediating role of research anxiety in the relationship between research stress and research performance. This provides a more comprehensive theoretical explanation of how research-related demands are associated with occupational outcomes among healthcare professionals. Notably, while many studies have explored the effects of general work stress on occupational health, relatively few have specifically investigated the impact of research-related stress on healthcare professionals’ psychological well-being and performance outcomes. In particular, the mediating role of research anxiety has rarely been examined. By addressing this gap, the present study broadens the research perspective on occupational stress among healthcare professionals and highlights the unique implications of research-related stressors.

### 4.3 Implications for clinical practice

The findings of this study have important implications for clinical practice and healthcare management. First, given that research stress is significantly associated with research anxiety and job satisfaction, hospital administrators should pay closer attention to reducing research-related pressure, especially in contexts where demanding clinical responsibilities coexist with research obligations. Hospitals may consider providing additional research resources, establishing dedicated research support teams, and optimizing work schedules to help healthcare professionals better manage research demands and enhance research performance. Second, the mediating role of research anxiety suggests that psychological factors are critical in understanding the impact of research stress. Healthcare institutions should incorporate mental health support into research training programs. For example, mindfulness-based interventions and stress management training may help healthcare professionals regulate anxiety, improve research confidence, and enhance work efficiency. Such interventions may not only improve research performance but also reduce burnout and increase overall job satisfaction (37, 38). Furthermore, as research performance was found to be significantly associated with job satisfaction, hospitals and research institutions should prioritize the development of research competencies and academic productivity among healthcare professionals. Encouraging research participation and recognizing research achievements can enhance professionals’ sense of accomplishment and social recognition. By promoting research capacity and achievement, healthcare institutions may strengthen professional identity, improve job satisfaction, and ultimately enhance the overall quality of healthcare services.

### 4.4 Limitations

Although this study provides important insights into the relationships among research stress, research anxiety, research performance, and job satisfaction, several limitations should be acknowledged. First, the use of convenience sampling may limit the representativeness of the sample. Future studies could employ multicenter random sampling strategies and recruit larger, more diverse samples to enhance the generalizability of the findings. Second, this study relied on self-reported measures to assess research stress, research anxiety, and related variables, which may be subject to social desirability bias. Participants might have provided responses that aligned with perceived social expectations. Future research could adopt multi-method approaches, such as combining survey data with qualitative interviews or physiological indicators, to improve data accuracy and reliability. Third, the cross-sectional design limits the ability to draw causal inferences. Fourth, this study did not conduct subgroup analyses by occupation or region due to limited sample sizes in certain subgroups. Future research could explore these differences with larger, stratified samples. Although path analysis revealed significant associations among the variables, longitudinal studies are needed to clarify the causal relationships between research stress and job satisfaction and to examine how research anxiety may evolve over time.

## 5 Conclusion

This study elucidates the complex relationships among research stress, research anxiety, research performance, and job satisfaction among healthcare professionals. The findings highlight the negative association of research stress with job satisfaction, particularly through the mediating role of research anxiety. In addition, strong research performance was found to be significantly associated with higher job satisfaction. Therefore, while improving the research environment, hospitals and healthcare institutions should prioritize psychological well-being and provide adequate research support and targeted psychological interventions. Such efforts may help reduce research-related stress and anxiety, enhance research performance, and ultimately improve job satisfaction among healthcare professionals. The findings provide theoretical support for hospital administrators in developing more effective intervention strategies and offer valuable insights for future development in the healthcare field.

## Data Availability

The raw data supporting the conclusions of this article will be made available by the authors, without undue reservation.
