# Supplementary material for: A study on the relationship between research stress, research anxiety, research performance, and job satisfaction among Chinese healthcare professionals and its influencing mechanisms: a national multi-center survey
Source: Front Psychiatry. 2026 Jul 16;17:1894224. doi: 10.3389/fpsyt.2026.1894224 (PMC13422427; doi:10.3389/fpsyt.2026.1894224)
Supplement: Supplementary file 1 [file DataSheet1.pdf]

**Supplementary Table 1 Cronbach Reliability and Convergent Validity Analysis**

| Scale | Dimension | Specific Content | Cronbach α | AVE |
|---|---|---|---|---|
| Job Satisfaction, JS | JS | JS1: Medicine is an ideal career for a lifetime.<br>JS2: If I had the chance to choose again, I would still choose to study medicine.<br>JS 3: I am very satisfied with my current job.<br>JS 4: I am considering changing careers or pursuing a second degree in the future.<br>JS 5: I regret choosing medicine as my career.<br>JS 6: Compared to medicine, I would prefer another job with the same salary. | 0.899 | 0.599 |
| Research Stress, RS | Task Pressure, TP | RS 1: The research team I am in has very strict requirements for our work and sets high goals.<br>RS 2: The number of research tasks I need to complete exceeds the range that I can easily cope with.<br>RS 3: I often feel that the time for research work is very tight, with hardly any spare time.<br>RS 4: I bear very key roles and responsibilities in the team, which brings great pressure. | 0.872 | 0.632 |
| | Innovation Pressure, IP | RS 5: I find it very difficult to identify novel and challenging research topics.<br>RS 6: During the research process, I often feel that it is very difficult to achieve innovative research results.<br>RS 7: Publishing high-level academic papers always presents numerous difficulties and is a very challenging process. | 0.808 | 0.585 |
| | Cooperation and Communication Pressure, CCP | RS 8: It is difficult for me to find partners who share my research interests, and cooperation is challenging.<br>RS 9: The task allocation in my research group is often chaotic, lacking clear guidance and collaboration.<br>RS 10: When communicating within the team, I often feel that my opinions are not sufficiently valued or understood, leading to poor cooperation outcomes. | 0.848 | 0.65 |
| | Resource Pressure and Career Development, RPCD | RS 11: When conducting academic research, I often lack sufficient resource support, such as funding or equipment.<br>RS 12: I feel that my academic career development has stagnated and there seems to be no room for progress.<br>RS 13: I often feel unable to establish an effective network within the academic community, which limits my career development. | 0.816 | 0.598 |
| Research Anxiety，RA | RA | RA 1: Before the research task is completed, I always worry that I can't meet the expected goal and feel a lot of pressure.<br>RA 2: During the research task, I often feel dissatisfied with the | 0.856 | 0.599 |

| | | | | |
|---|---|---|---|---|
| | | strict evaluation standards and think that I can't meet the requirements.<br>RA 3: Before the task is completed, I often feel powerless and frustrated, as if I am constantly failing.<br>RA 4: When the research task is not completed, I am very nervous and anxious, and I can hardly concentrate. | | |
| Research Performance，RP | RP | RP 1: I focus on publishing research papers of academic value.<br>RP 2: I always conduct research with a rigorous attitude.<br>RP 3: I have published several influential academic papers.<br>RP 4: I have received several academic awards for my research contributions. | 0.821 | 0.534 |

Job Satisfaction, JS; Research Stress, RS; Research Anxiety, RA; Research Performance, RP; Task Pressure, TP; Innovation Pressure, IP; Cooperation and Communication Pressure, CCP; Resource Pressure and Career Development, RPCD

**Supplementary Table 2 Table of Variance Explained**

| Factor Number | Eigenvalue | | | Variance Explained Before Rotation | | | Variance Explained After Rotation | | |
|---|---|---|---|---|---|---|---|---|---|
| | Eigenvalue | Variance Explained (%) | Cumulative (%) | Eigenvalue | Variance Explained (%) | Cumulative (%) | Eigenvalue | Variance Explained (%) | Cumulative (%) |
| 1 | 8.906 | 32.985 | 32.985 | 8.906 | 32.985 | 32.985 | 4.077 | 15.099 | 15.099 |
| 2 | 2.659 | 9.85 | 42.834 | 2.659 | 9.85 | 42.834 | 2.95 | 10.924 | 26.024 |
| 3 | 1.97 | 7.297 | 50.131 | 1.97 | 7.297 | 50.131 | 2.798 | 10.362 | 36.385 |
| 4 | 1.614 | 5.976 | 56.107 | 1.614 | 5.976 | 56.107 | 2.675 | 9.908 | 46.293 |
| 5 | 1.423 | 5.271 | 61.379 | 1.423 | 5.271 | 61.379 | 2.255 | 8.353 | 54.646 |
| 6 | 1.339 | 4.959 | 66.338 | 1.339 | 4.959 | 66.338 | 2.137 | 7.915 | 62.561 |
| 7 | 1.107 | 4.101 | 70.439 | 1.107 | 4.101 | 70.439 | 2.127 | 7.878 | 70.439 |
| 8 | 0.59 | 2.185 | 72.624 | - | - | - | - | - | - |
| 9 | 0.54 | 2.002 | 74.626 | - | - | - | - | - | - |
| 10 | 0.515 | 1.909 | 76.535 | - | - | - | - | - | - |
| 11 | 0.493 | 1.826 | 78.361 | - | - | - | - | - | - |
| 12 | 0.481 | 1.783 | 80.143 | - | - | - | - | - | - |
| 13 | 0.464 | 1.718 | 81.861 | - | - | - | - | - | - |
| 14 | 0.43 | 1.592 | 83.454 | - | - | - | - | - | - |
| 15 | 0.418 | 1.548 | 85.002 | - | - | - | - | - | - |
| 16 | 0.407 | 1.507 | 86.509 | - | - | - | - | - | - |
| 17 | 0.388 | 1.438 | 87.946 | - | - | - | - | - | - |
| 18 | 0.37 | 1.37 | 89.316 | - | - | - | - | - | - |
| 19 | 0.364 | 1.35 | 90.666 | - | - | - | - | - | - |
| 20 | 0.36 | 1.334 | 92 | - | - | - | - | - | - |

| | | | | | | | | | |
|---|---|---|---|---|---|---|---|---|---|
| 21 | 0.343 | 1.271 | 93.271 | - | - | - | - | - | - |
| 22 | 0.339 | 1.255 | 94.525 | - | - | - | - | - | - |
| 23 | 0.321 | 1.188 | 95.713 | - | - | - | - | - | - |
| 24 | 0.313 | 1.158 | 96.871 | - | - | - | - | - | - |
| 25 | 0.291 | 1.077 | 97.948 | - | - | - | - | - | - |
| 26 | 0.282 | 1.044 | 98.992 | - | - | - | - | - | - |
| 27 | 0.272 | 1.008 | 100 | - | - | - | - | - | - |

**Supplementary Table 3 rotated Factor Loadings**

| Item | Factor Loadings Coefficient | | | | | | |
|---|---|---|---|---|---|---|---|
| | Factor 1 | Factor 2 | Factor 3 | Factor 4 | Factor 5 | Factor 6 | Factor 7 |
| JS1 | **0.77** | -0.104 | -0.139 | 0.151 | -0.071 | -0.075 | -0.141 |
| JS2 | **0.812** | -0.101 | -0.156 | 0.055 | -0.053 | -0.073 | -0.097 |
| JS3 | **0.794** | -0.092 | -0.137 | 0.076 | -0.081 | -0.064 | -0.069 |
| JS4 | **0.772** | -0.043 | -0.118 | 0.116 | -0.108 | -0.147 | -0.064 |
| JS5 | **0.761** | -0.128 | -0.113 | 0.109 | -0.092 | -0.087 | -0.062 |
| JS6 | **0.776** | -0.065 | -0.114 | 0.14 | -0.084 | -0.043 | -0.028 |
| RS1 | -0.094 | **0.789** | 0.091 | -0.117 | 0.181 | 0.096 | 0.137 |
| RS2 | -0.131 | **0.812** | 0.089 | -0.129 | 0.113 | 0.155 | 0.102 |
| RS3 | -0.132 | **0.817** | 0.104 | -0.098 | 0.124 | 0.126 | 0.143 |
| RS4 | -0.112 | **0.753** | 0.144 | -0.063 | 0.177 | 0.183 | 0.124 |
| RS5 | -0.134 | 0.189 | 0.148 | -0.107 | 0.116 | **0.733** | 0.191 |
| RS6 | -0.115 | 0.189 | 0.104 | -0.106 | 0.136 | **0.812** | 0.103 |
| RS7 | -0.156 | 0.155 | 0.138 | -0.133 | 0.121 | **0.775** | 0.184 |
| RS8 | -0.147 | 0.232 | 0.116 | -0.098 | **0.787** | 0.143 | 0.178 |
| RS9 | -0.135 | 0.188 | 0.129 | -0.104 | **0.818** | 0.11 | 0.108 |
| RS10 | -0.134 | 0.162 | 0.096 | -0.126 | **0.806** | 0.126 | 0.162 |
| RS11 | -0.139 | 0.135 | 0.099 | -0.15 | 0.163 | 0.204 | **0.782** |
| RS12 | -0.13 | 0.212 | 0.144 | -0.181 | 0.109 | 0.115 | **0.761** |
| RS13 | -0.113 | 0.156 | 0.173 | -0.105 | 0.189 | 0.175 | **0.768** |
| RA1 | -0.187 | 0.085 | **0.799** | -0.105 | 0.066 | 0.15 | 0.114 |
| RA2 | -0.191 | 0.084 | **0.767** | -0.145 | 0.11 | 0.074 | 0.109 |
| RA3 | -0.156 | 0.115 | **0.798** | -0.147 | 0.075 | 0.077 | 0.08 |
| RA4 | -0.174 | 0.139 | **0.75** | -0.177 | 0.108 | 0.11 | 0.117 |
| RP1 | 0.156 | -0.105 | -0.109 | **0.755** | -0.13 | -0.042 | -0.104 |
| RP2 | 0.107 | -0.106 | -0.133 | **0.791** | -0.077 | -0.083 | -0.079 |
| RP3 | 0.161 | -0.092 | -0.14 | **0.737** | -0.041 | -0.101 | -0.129 |
| RP4 | 0.111 | -0.072 | -0.146 | **0.767** | -0.073 | -0.111 | -0.092 |

Rotation method: Varimax with the goal of maximizing variance.

**Supplementary Table 4: Factor Loading Table**

| Items | | | Estimate | S.E. | C.R. | P | STD Estimate |
|---|---|---|---|---|---|---|---|
| JS1 | <--- | JS | 1 | | | | 0.787 |
| JS2 | <--- | JS | 1.019 | 0.039 | 26.226 | *** | 0.808 |
| JS3 | <--- | JS | 0.971 | 0.039 | 25.074 | *** | 0.778 |
| JS4 | <--- | JS | 0.939 | 0.038 | 24.557 | *** | 0.765 |
| JS5 | <--- | JS | 0.916 | 0.038 | 24.094 | *** | 0.753 |
| JS6 | <--- | JS | 0.898 | 0.037 | 23.985 | *** | 0.75 |
| RS1 | <--- | TP | 1 | | | | 0.779 |
| RS2 | <--- | TP | 1.037 | 0.041 | 25.177 | *** | 0.809 |
| RS3 | <--- | TP | 1.066 | 0.042 | 25.581 | *** | 0.822 |
| RS4 | <--- | TP | 0.958 | 0.04 | 23.76 | *** | 0.768 |
| RS5 | <--- | IP | 1 | | | | 0.733 |
| RS6 | <--- | IP | 1.052 | 0.051 | 20.519 | *** | 0.768 |
| RS7 | <--- | IP | 1.083 | 0.052 | 20.901 | *** | 0.792 |
| RS8 | <--- | CCP | 1 | | | | 0.831 |
| RS9 | <--- | CCP | 0.937 | 0.037 | 25.107 | *** | 0.796 |
| RS10 | <--- | CCP | 0.946 | 0.038 | 24.955 | *** | 0.791 |
| RS11 | <--- | RPCD | 1 | | | | 0.788 |
| RS12 | <--- | RPCD | 0.962 | 0.044 | 21.666 | *** | 0.748 |
| RS13 | <--- | RPCD | 0.998 | 0.044 | 22.46 | *** | 0.782 |
| RA1 | <--- | RA | 1 | | | | 0.797 |
| RA2 | <--- | RA | 0.986 | 0.042 | 23.324 | *** | 0.756 |
| RA3 | <--- | RA | 0.966 | 0.04 | 23.9 | *** | 0.773 |
| RA4 | <--- | RA | 0.991 | 0.042 | 23.777 | *** | 0.77 |
| RP1 | <--- | RP | 1 | | | | 0.725 |
| RP2 | <--- | RP | 1.045 | 0.052 | 20.169 | *** | 0.758 |
| RP3 | <--- | RP | 0.981 | 0.051 | 19.211 | *** | 0.714 |
| RP4 | <--- | RP | 0.995 | 0.051 | 19.504 | *** | 0.727 |

* $p<0.05$ ** $p<0.01$ *** $p<0.001$

**Supplementary Table 5 Correlation Analysis**

| | JS | RS | RA | RP |
|---|---|---|---|---|
| JS | 1 | | | |
| RS | -0.430*** | 1 | | |
| RA | -0.422*** | 0.470*** | 1 | |

| | | | | |
|---|---|---|---|---|
| RP | 0.354*** | -0.439*** | -0.403*** | 1 |

*** $p<0.01$

**Supplementary Table 6 Structural Equation Model Fit Index Analysis**

| Indicator | CMIN/DF | RMR | GFI | NFI | TLI | CFI | RMSEA |
|---|---|---|---|---|---|---|---|
| Optimal Indicator | <3 | <0.05 | >0.9 | >0.9 | >0.9 | >0.9 | <0.08 |
| Acceptable Indicator | <5 | <0.08 | >0.8 | >0.8 | >0.8 | >0.8 | <0.1 |
| Measurement Result | 1.569 | 0.028 | 0.962 | 0.961 | 0.984 | 0.986 | 0.025 |
